# An overview of technologies for immobilization of enzymes and surface analysis techniques for immobilized enzymes

**DOI:** 10.1080/13102818.2015.1008192

**Published:** 2015-02-17

**Authors:** Nur Royhaila Mohamad, Nur Haziqah Che Marzuki, Nor Aziah Buang, Fahrul Huyop, Roswanira Abdul Wahab

**Affiliations:** ^a^ Department of Chemistry, Faculty of Science, Universiti Teknologi Malaysia, Skudai81310, Johor, Malaysia; ^b^ Department of Biotechnology and Medical Engineering, Faculty of Bioscience and Medical Engineering, Universiti Teknologi Malaysia, Skudai81310, Johor, Malaysia

**Keywords:** enzymes, immobilization, entrapment, surface analysis, nanoscale, atomic force spectroscopy, circular dichroism

## Abstract

The current demands of sustainable green methodologies have increased the use of enzymatic technology in industrial processes. Employment of enzyme as biocatalysts offers the benefits of mild reaction conditions, biodegradability and catalytic efficiency. The harsh conditions of industrial processes, however, increase propensity of enzyme destabilization, shortening their industrial lifespan. Consequently, the technology of enzyme immobilization provides an effective means to circumvent these concerns by enhancing enzyme catalytic properties and also simplify downstream processing and improve operational stability. There are several techniques used to immobilize the enzymes onto supports which range from reversible physical adsorption and ionic linkages, to the irreversible stable covalent bonds. Such techniques produce immobilized enzymes of varying stability due to changes in the surface microenvironment and degree of multipoint attachment. Hence, it is mandatory to obtain information about the structure of the enzyme protein following interaction with the support surface as well as interactions of the enzymes with other proteins. Characterization technologies at the nanoscale level to study enzymes immobilized on surfaces are crucial to obtain valuable qualitative and quantitative information, including morphological visualization of the immobilized enzymes. These technologies are pertinent to assess efficacy of an immobilization technique and development of future enzyme immobilization strategies.

## Introduction

One of the most important roles of enzymes as natural biocatalysts is their capacity to enhance the rate of virtually all chemical reactions within a cell. Enzymes increase the rates of chemical reactions without themselves being permanently altered or consumed by the reactions. They also increase the reaction rates without changing the equilibrium between the reactants and the products.[1] The rates of the reactions are speeded up by well over a million-fold, so reactions that take years to complete in the absence of catalysis can now occur within fractions of a second, when in the presence of the appropriate enzyme. In the absence of enzymes, progress of most biochemical reactions will be significantly slowed down, so that they would no longer be able to sustain complex life. Nevertheless, the use of enzyme is usually associated with other drawbacks resulting from sensitivity to process conditions, low stability or from propensity to be inhibited by high concentrations of reaction components.[2,3] The majority of enzymes are fairly unstable and industrial application is often hampered by a lack of long-term operational stability and the technically challenging recovery process and reuse of the enzyme.[4,5] Also, a protein's sequence and interactions between residues in the protein core are naturally not fully optimized and only achieve the minimum requirements for proper functioning. This situation leaves plenty of room for improvement.[6–8]

In order to make enzyme utilization in biotechnological processes more favourable, different methods for cost reduction have been put into practice and, immobilization is one of them. The term ‘immobilized enzymes’ refers to ‘enzymes physically confined or localized in a certain defined region of space with retention of their catalytic activities, and which can be used repeatedly and continuously.’[9] Besides a more convenient handling of the enzyme, it also substantially simplifies the manipulation with the biocatalyst and the control of the reaction process [3] while enhancing the stability of the enzyme under both storage and operational conditions. Immobilization provides a facile separation of the enzyme from the product,[5,10,11] hence protein contamination of the product is minimized or avoided altogether. Apart from easy separation of the enzyme from the reaction mixture, enzyme immobilization also remarkably reduces the cost of enzyme and the enzymatic products. Insolubilization of the enzyme by attachment to a matrix also imparts several added benefits such as (1) rapid arrest of the reaction by removal of the enzyme from the reaction solution and (2) improvement of enzyme stability against temperature, solvents, pH, contaminants and impurities.[2,5,11–13] It also helps for efficient recovery and reuse of expensive enzymes [5,13,14] and permits their application in continuous fixed-bed operation.[5,15] It is possible to conclude that enzyme immobilization increases the productivity of the biocatalysts and enhances their features, making them more attractive for diverse applications.

The principal components of an immobilized enzyme system are the enzyme, the matrix and the mode of attachment. The driving forces for enzyme immobilization are the improvement of enzyme stability, increment of volume specific enzyme loading and simplification of biocatalyst recycling and downstream processing.[16] The immobilization methods exploit the fact that proteins have amino acids with different features,[5,17] whereby functional groups in side chains of these amino acids can be involved in binding to the support through various types of linkages and interactions. The enzymes can be attached by interactions ranging from reversible physical adsorption, ionic linkages and affinity binding, to the irreversible but stable covalent bonds that are present through ether, thio-ether, amide or carbamate bonds.[17] In this review, we highlight the principal factors in the development of immobilized biocatalysts which include the selection of immobilization supports, conditions and methods with respect to activity and stability of the immobilized enzymes. We also discuss the various surface analytical techniques used to quantify enzyme attachment on the surface of the carrier as well the parameters that are evaluated for the immobilized enzymes. Detail descriptions of the surface properties of immobilized enzymes are mandatory as these parameters are used to compare and assess the efficacy of the different biocatalyst preparations.

## Factors to consider prior to enzyme immobilization

It is important to recognize that an enzyme would undergo changes in the chemical and physical properties upon immobilization, depending on the choice of immobilization method. The changes of the microenvironment imposed upon them by the supporting matrix and by the products of their own action have been observed to alter the stability of enzymes and also their kinetic properties. The surface on which the enzyme is immobilized has several fundamental roles to play such as maintaining the tertiary structure of the enzyme by formation of electron transition complexes or by forming hydrogen or covalent bonds with the matrix.[18] Hence, the key consideration when immobilizing an enzyme on a surface is the proper selection of an attachment method between the reactive groups on the matrix surface and the residues outside the substrate binding or active site of the enzyme.[19] Preservation of the catalytically active tertiary structure of the enzyme is also an essential factor in maximizing stability and reactivity of the enzyme in its immobilized state.[20] Hence, there are three fundamental factors to be considered in the development of immobilized biocatalysts: selection of immobilization supports, conditions and methods of immobilization.

### Choice of supports

The characteristics of the matrix are paramount in determining the effectiveness of the immobilized enzyme system. The selection of the optimum support material can affect the immobilization process whereby properties of both the enzyme and support material will dictate the properties of the supported enzyme preparation. Hence, the interaction between the two confers an immobilized enzyme with specific mechanical, chemical, biochemical and kinetic properties.[5] Ideal support properties are described to include hydrophilicity, inertness towards enzymes, biocompatibility, resistance to microbial attack, resistance to compression and readily accessible at a low cost.[21–23]

Even though there is no universal support that is appropriately suited for all enzymes and applications, certain characteristics of the support material should be considered such as having high affinity for protein, availability of reactive functional group, mechanical stability, rigidity, feasibility of regeneration, non-toxicity and biodegradability.[24] However, the use of common materials for enzyme immobilization namely, silica-based carriers, acrylic resins, synthetic polymers, active membranes and exchange resins faces drawbacks such as high cost of materials and technology necessary to apply fixation methods which greatly increases the costs of biocatalyst.

Various immobilization methods and supports have been developed in order to improve enzyme activity.[25,26] The selection of support material can be a rather complex matter since it depends on the type of enzyme, reaction media, safety policy in the field of hydrodynamic conditions and reaction conditions.[19,27,28] Different supports offer variability in their physical and chemical properties (e.g. pore size, hydrophilic/hydrophobic balance and surface chemistry) for enzyme attachment. Their differences in morphological and physical characteristics can affect enzyme immobilization and its catalytic properties,[29,30] since the support is directly contacted to the enzyme.[31]

Supports can be classified as organic and inorganic according to their chemical composition, and can be further subdivided into natural and synthetic polymers. The most commonly used supports are carboxymethyl-cellulose, starch, collagen, modified sepharose, ion exchange resins, active charcoal, silica,[32] clay,[33] aluminium oxide, titanium, diatomaceous earth, hydroxyapatite, ceramic, celite,[34–36] agarose, [17,37] or treated porous glass which is an organic material [38] and certain polymers.[39] One of the desired properties of support matrix is the mesoporous material where the large surface areas and greater number of pores will lead to higher enzyme loading per unit mass.[40] Porous supports are generally preferred as the high surface area permits a higher enzyme loading and the immobilized enzyme receives better protection from the environment. The pore parameters and particle size of the support establish the total surface area and thus, critically affect the capacity for binding of enzymes. These supports should also have a controlled pore distribution in order to optimize capacity and flow properties.[2] For example, vesicular silica or vesicle-like mesoporous material which possesses curved interlamellar, mesochannel and multimellar structure enhances the affinity of interaction resulting in slower lipase leakage during the recycle process.[41]

Nowadays nanostructured forms such as nanoparticles, nanofibres, nanotubes and nanocomposites are preferred to be used as carrier for enzyme immobilization and stabilization,[10,42] even if it is costly. These robust nanoscaffolds are excellent support materials for enzyme immobilization as they have the ideal characteristics for balancing the key aspects that determine the efficiency of biocatalysts, for instance, inherently large surface area and high mechanical properties that allow effective enzyme amount with minimum diffusion limitation [10,11,43] as well as high volumetric enzyme.[44] The properties of carbon nanotubes also confer easy separation or reusability of the biocatalyst by simple filtration [10] or using a magnetic field.[45] Compared to the conventional immobilization techniques, nanoparticle-based immobilization provided three important attributes; (1) facile synthesis of nanoenzyme particles in high solid content without the use of surfactants and toxic reagents, (2) possible tailoring of particle size within effective working limits and (3) attainment of homogeneous and well-defined core-shell nanoparticles with a thick enzyme shell.[46] Also, with the growing attention given to cascade enzymatic reaction and *in vitro* synthetic biology, co-immobilization of multienzymes on these nanoparticles could also be accomplished.

## Techniques of enzyme immobilization

**Figure 1.**
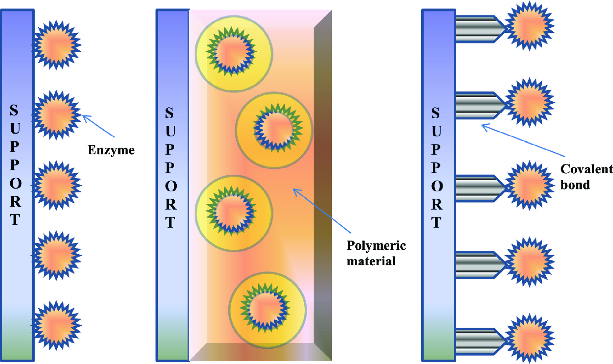
Schematics of the three most common enzyme immobilization techniques: (A) physical adsorption, (B) entrapment and (C) covalent attachment/cross-linking.[27]

Selection of the appropriate immobilization method is a very crucial part of the immobilization process as it plays the biggest role in determining the enzyme activity and characteristics in a particular reaction. Process specifications for the catalyst, including overall enzymatic activity, effectiveness of the lipase utilization, enzyme deactivation and regeneration characteristics, cost of immobilization procedure, toxicity of immobilization reagents and the desired final properties of the immobilized enzymes are factors that should be considered.[47] Basically immobilization methods can be divided into two general classes namely, the chemical and physical methods. Physical methods are characterized by weaker, monocovalent interactions such as hydrogen bonds, hydrophobic interactions, van der Waals forces, affinity binding, ionic binding of the enzyme with the support material, or mechanical containment of enzyme within the support.[17,48] In the chemical method, formation of covalent bonds achieved through ether, thio-ether, amide or carbamate bonds [17] between the enzyme and support material are involved.[47] There are four principal techniques for immobilization of enzymes namely, adsorption, entrapment, covalent and cross-linking (Figure 1). However, not one method is ideal for all molecules or purposes considering the inherently complex nature of the protein structure.

### Physical adsorption

The physical adsorption method can be defined as one of the straightforward methods of reversible immobilization that involve the enzymes being physically adsorbed or attached onto the support material. Adsorption can occur through weak non-specific forces such as van der Waals, hydrophobic interactions and hydrogen bonds,[49–51] whereas in ionic bonding the enzymes are bound through salt linkages. The reversibly immobilized enzymes can be removed from the support under gentle conditions, a method highly attractive as when the enzymatic activity has decayed, the support can be regenerated and reloaded with fresh enzyme. This is because of economic reasons as the cost of the support is often a primary factor in the overall cost of immobilized catalysts.[17,52,53]

Physical adsorption usually requires soaking of the support into a solution of the enzyme and incubating to allow time for the physical adsorption to occur. Another way is allowing a solution of the enzyme to dry on the electrode surfaces and then rinsing away enzymes that are not adsorbed.[54,55] However, these relatively weak non-specific forces suffer from drawbacks such as enzyme leakage from the matrix. As for enzyme immobilization through purely ionic forces between the enzyme and support, it is based on the protein–ligand interaction principles used in chromatography, namely the reversible immobilization of enzymes which was first used in ion exchangers.[9] Depending on the pH of the solution and the isoelectric point, the surface of the enzyme may bear charges [56] and its charge distribution can be readily calculated and displayed using current available modelling systems.[57] Any ion exchanger can act as carrier in immobilization via ionic and strongly polar interactions. The use of immobilized polymeric-ionic ligands has allowed for modulation of protein–matrix interactions and optimization of the derivative properties.[58] However, the highly charged support as well as substrates or products could present other problems such as distortion of kinetics due to partitioning or diffusion phenomena, and subsequently alter the pH stability or pH optimum of the enzyme.[36]

In some cases, affinity binding is also included as one of the physical methods for immobilization of enzymes.[2,38] The principle of affinity binding exploits the selectivity between complementary biomolecules for application in enzyme immobilization. The remarkable selectivity of the interaction, control orientation of immobilized enzyme and minimal conformational changes caused by this type of binding resulting in high retention of the immobilized molecule activity are key advantages of the method [17,48] (for instance, the binding between antibodies and antigens or haptens, lectins and free saccharidic chains or glycosylated macromolecules, nucleic acids and nucleic acid-binding proteins, hormones and their receptors, avidin and biotin, polyhistidine tag and metal ions, etc.).[59,60] Affinity binding between enzyme and support is achieved in two ways: either the support is pre-coupled to an affinity ligand for target enzyme or the enzyme is conjugated to an entity that develops affinity towards the support.[36,61] Further modification of the affinity binding method is bioaffinity layering that exponentially increases enzyme-binding capacity and reusability through various interactions such as Coulombic, hydrogen bonding and van der Waals forces.[36,62]

Entropically driven hydrophobic interactions are also used to bind enzymes to the surfaces of the support. When one enzyme molecule displaces a large number of water molecules both from the support and its own surface during immobilization, it results in entropy gain to produce the hydrophobic interactions between both entities.[57] The strength of the interactions depends on the hydrophobicity of both the adsorbent and protein, regulated by the size of the hydrophobic ligand molecule and the degree of substitution of the support. Further modulation of the hydrophobic interactions between the enzyme and support is achieved through adjustment of the pH, temperature and concentration of salt during enzyme immobilization.[17,63]

In general, enzyme immobilization through the technique of physical adsorption is quite simple and may have a higher commercial potential due to its simplicity, low cost and retaining high enzyme activity [64] as well as a relatively chemical-free enzyme binding.[65] In most cases, the cell productivity is not affected but tactlessly such method has few disadvantages for instance, quite liable to changes under certain conditions such as pH, temperature and ionic strength of the buffer.[2,51,66] Physical bonding is generally too weak to keep the enzyme fixed to the carrier and is prone to leaching of the enzyme.[67,68] Enzyme leaching can be further enhanced when subjected to industrial conditions of high reactant and product concentrations and high ionic strength [5] thereby, potential contamination of the substrate.

### Entrapment

Entrapment is defined as an irreversible method of enzyme immobilization where enzymes are entrapped in a support or inside of fibres, either the lattice structure of a material or in polymer membranes [69–71] that allows the substrate and products to pass through but retains the enzyme.[14,48] Entrapment is also described as physical restriction of enzyme within a confined space or network.[72] Typically, entrapment can improve mechanical stability and minimize enzyme leaching [73] and the enzyme does not chemically interact with the polymer; therefore, denaturation is usually avoided. The method permits the ability to modify the encapsulating material and hopefully create an optimal microenvironment for the enzyme (i.e. matching the physico-chemical environment of the enzyme and immobilization material). The ideal microenvironment could be optimal pH, polarity or amphilicity. This can be achieved with a variety of materials including polymers, sol-gels, polymer/sol-gel composites and other inorganic materials.[36,74–77]

The relation between support material pore size and adsorption is that the adsorption can be done only externally if pores are too small and vice versa.[78] Gelation of polyanionic or polycationic polymers by the addition of multivalent counter-ions is the simplest and common method of enzyme entrapment. It is possible to use the following polymers as a matrix: alginate, carrageenan, collagen, polyacrylamide, gelatin, silicon rubber, polyurethane and polyvinyl alcohol with styrylpyridium group.[2,79] Alginates, however, are one of the most frequently used polymers due to their mild gelling properties and non-toxicity.[72] Nonetheless, the practical use of this method is rather limited as it tends to incur mass transfer limitations of substrate or analyte to the enzyme active site.[38] Other disadvantages include possibility of enzyme leakage [5] which can occur when the pores of the support matrix are too large, deactivation during immobilization, low loading capacity and abrasion of support material during usage. Also, the ratio of immobilized particle size to the support material pore size is a significant factor to be considered for the usability of ready probes.[38]

### Cross-linking

Cross-linking is another irreversible method of enzyme immobilization that does not require a support to prevent enzyme loss into the substrate solution.[40,69,80,81] The method is also called carrier-free immobilization [5] where the enzyme acts as its own carrier and virtually pure enzyme is obtained eliminating the advantages and disadvantages associated with carriers.[5,57] The use of carrier inevitably leads to dilution of activity owing to the introduction of large portion of non-catalytic ballast. The percentage of ballast used can range from 90% to over 99%, resulting in low space-time yields [5,30,82] as well as being costly.[82]

Technically, cross-linking is performed by formation of intermolecular cross-linkages between the enzyme molecules by means of bi- or multifunctional reagents. The most commonly used cross-linking reagent is glutaraldehyde as it is economical and easily obtainable in large quantities.[5,38,57] It has been used for decades for cross-linking proteins via reaction of the free amino groups of lysine residues, on the surface of neighbouring enzyme molecules, with oligomers or polymers of glutaraldehyde resulting from inter- and intramolecular aldol condensations. Cross-linking can involve both Schiff's base formation and Michael-type 1,4 addition to *α*,*β*-unsaturated aldehyde moieties, and the exact mode of cross-linking is pH dependent.[82,83]

Cross-linked enzyme aggregates (CLEAs) are first prepared by aggregating the enzymes in precipitants such as acetone, ammonium sulphate and ethanol followed by a cross-linker,[57] and the reactions for enzyme immobilization can be executed in three different manners; either by mixing the prepolymers with a photosensitizer (e.g. benzoin ethyl ether), melting, mixing with an enzyme solution, and gelling by exposure to near ultraviolet (UV) radiation, or freezing the enzyme-containing monomer solution in the form of small beads. This is followed by polymerization initiated by gamma radiation, or the enzymes are mixed in a buffered aqueous solution containing acrylamide monomer and a cross-link agent before a chemically initiated polymerization is performed.[84] Recently, nanodiametric supports have brought a decisive change in the field of biocatalyst immobilization.[33,36,64,85–87] Cross-linking of enzymes to electrospun nanofibres has shown better residual activity due to increased surface area and porosity. Lysozyme-immobilized electrospun Chitosan (CS) nanofibres via CLEAs have also been reported to be effective in continuous antibacterial applications.[88]

### Covalent bonding

Covalent bonding is one of the most widely used methods for irreversible enzyme immobilization. The functional group that takes part in the binding of the enzyme usually involves binding via the side chains of lysine (ϵ-amino group), cysteine (thiol group) and aspartic and glutamic acids (carboxylic group,[2,89] imidazole and phenolic groups which are not essential for the catalytic activity of enzyme).[84] Activity of the covalent bonded enzyme depends on the size and shape of carrier material, nature of the coupling method, composition of the carrier material and specific conditions during coupling.[84]

For the covalent attachment between enzyme and support, the direction of the enzyme binding is a crucial factor that determines its stability. It has been reported that the highest enzyme activity level is achieved when the active centre amino acids is not involved in the binding with the support. The coupling with the support can be done in two ways, depending on active groups present in the molecule that is to be immobilized. The reactive functional groups can be added to the support without modifications, or the support matrix is modified to generate activated groups. In both cases, it is anticipated that the electrophilic groups generated on the support will react with strong nucleophiles on the protein. Matrices of choice for such interactions usually include agarose, cellulose, poly(vinyl chloride), ion exchange resins and porous glass. Dandavate [90] and Yilmaz and co-workers [91] covalently immobilized *Candida rugosa* lipase onto the surface of silica nanoparticles and glutaraldehyde-activated aminopropyl glass beads which resulted in easy recovery and reuse of the enzyme for ester synthesis. Similarly, Damnjanovíc and co-workers reported that covalently bound *Candida rugosa* lipase was a robust and versatile biocatalyst for production of aroma ester in a fluidized bed reactor.[92]

Covalent bonds provide powerful link between the lipase and its carrier matrix, allow its reuse more often than with other available immobilization methods [5,93] and prevent enzyme release into the reaction environment.[2,49,84] The method also increases half-life and thermal stability of enzymes when coupled with different supports like mesoporous silica, chitosan, etc.[94] The conferred stability comes from unlimited covalent binding between the enzyme and substrate due to the lack of any barrier between them. Localization of the enzyme on the surface of the support further enhances enzyme attachment and enzyme loading binding method.[95]

## Surface analysis technology for enzyme immobilization

The orientation and three-dimensional structure of immobilized enzymes are crucial to ensure high enzyme stability and activity. Most immobilization procedures do not actively control the orientation of the enzymes, resulting in the inevitable burying and inaccessibility of their active site. Enzymes undergo substantial changes in the surface microenvironment, conformation and protein refolding following an immobilization process. This could explain the dramatically diminished activity and stability which is often observed when an enzyme is immobilized on a surface.[96] Hence, formation of individual biomolecules, their orientation on surfaces and their functionality, as well as the homogeneity of the surface coverage could be the key for solving many challenging questions in the biosensor field and in the development of sophisticated biocatalyst for wide biotechnological applications.

### Thermal gravimetric analysis (TGA)

Thermal gravimetric analysis (TGA) is a thermal analysis technique that is defined as thermal analysis of ‘a group of techniques in which a physical property of a substance and/or its reaction products is measured as a function of temperature, while the substance is subjected to a controlled temperature program.’[97] The mass of a sample in a controlled atmosphere is measured as a function of temperature or time. TG may be used to monitor any reaction that involves a gaseous phase, such as oxidation or dehydration. The sample size varies from a few mg to 10 g depending on the equipment used. The method measures a sample's weight as it is heated or cooled in a furnace and is widely used to characterize and verify materials. In the thermograms weight versus temperature or time helps to generate information about thermal stability of the sample, quantitative determination of components, reaction rates, oxidation and kinetics of decomposition.[98,99] TGA is applicable to most industries and also useful in environmental, food science, biotechnology, pharmaceutical and petrochemical applications.

In previous studies, TGA is usually used to characterize the change in thermal stability of the support used in the immobilization process, either it has been modified or unmodified by measuring the decomposition or weight loss of the sample.[99,100] TGA can be used to estimate the new structure of enzyme immobilized or the support of choice in enzyme immobilization studies.[64] It is also used to determine the amount of functional groups attached on the support, namely the degree of functionalization following an enzyme immobilization process [44] to confirm success [101] as well as efficacy of the enzyme immobilization method.

### Field emission scanning electron microscopy (FESEM)/scanning electron microscopy (SEM) and transmission electron microscopy (TEM)

Electron microscopy is the only technique available to obtain structural information on materials at nanometre scale resolution. The scanning electron microscopy (SEM)/field emission scanning electron microscopy (FESEM) provides surface information with limited information about the internal structure. SEM is an analytical technique in which an image is formed on a cathode ray tube whose raster is synchronized with the raster of a point beam of electrons scanned over the surface of a specimen. The device bounces electrons off the surface of a sample to produce an image. The technique provides two outstanding improvements over the optical microscope: it extends the resolution limits up to 30,000× or as high as 60,000×, and improve depth of field by an approximate factor of 300. The drive to improve SEM technology was the need for an FESEM capable of ultrahigh resolution over the entire accelerating voltage range by advances in secondary electron detector technology. It was also required for greater flexibility for a wider range of analytical applications. Both SEM and FESEM are the most highly implemented techniques to characterize the morphological surface of the enzyme as well as the support for immobilization. SEM samples are commonly used to observe the morphology to confirm success of enzyme immobilization,[102,103] while FESEM is used to visualize very small topographic or morphology details on the surface or entire or fractioned objects.[104–106]

Structural information can also be obtained through the use of transmission electron microscopy (TEM). Unlike FESEM, TEM provides only two-dimensional projections of the solid structure.[107] TEM works by transmitting electrons instead of light through a specimen. The device allows for a much higher resolution than can be obtained with a light microscope, allowing for the visualization of even a single column of atoms. The electrons are shot completely through the sample, using a tungsten filament to produce an electron beam in a vacuum chamber. The emitted electrons are accelerated through an electromagnetic field that also narrowly focuses the beam. The beam is then passed through the sample material, in which electrons that pass through the sample hit a phosphor screen, charge coupled device (CCD) or film and produce an image. Sample of lower density allows more electrons to get through and the image is brighter. A staining method was adopted from the positive staining method for electron microscopy for biological samples whereby a darker image is produced in areas where the sample is denser and therefore, fewer electrons pass through. The method can produce images with resolution down to 0.2 nm which is smaller than the size of most atoms, hence, revealing the true structural arrangement of atoms in the sample material.[108]

Nowadays, TEM has become even more important as the structural dimensions in many materials science applications are rapidly approaching the nanometre length scale and are beyond the spatial resolution limits of other methods. In enzyme immobilization, the distribution of enzyme onto the support material needs to be visualized as it is an important parameter related to the accessibility of the enzyme to the substrate.[109] Besides, TEM also provides information on particle size and morphology of sample immobilization,[110] leading to many reports on enzyme immobilization which uses TEM for characterization especially on nano or micro size of support such as carbon nanotubes,[44,111,112] micron-size magnetic beads [113] and nanoparticles.[114]

### X-ray photoelectron spectroscopy (XPS)

Another method of molecular analysis for immobilized enzymes is X-ray photoelectron spectroscopy (XPS), also called electron spectroscopy for chemical analysis. XPS provides a total elemental analysis, except for hydrogen and helium at 10–200 Å, depending on the sample and instrumental conditions of any solid surface. The working of XPS is based on the photoelectric effect whereby each atom on the surface has core electron with the characteristic binding energy that is conceptually but not strictly equal to the ionization energy of that electron. The technique uses X-ray to probe the energy distribution of electrons ejected from the solid surface and the photoelectric effect: the electrons contain information regarding chemical oxidation state and electronic structure. The surface of the sample is irradiated with a low-energy X-ray which excites the electrons of the sample atoms. If the binding energy is lower than the X-ray energy, the electrons are then emitted from the parent atom as a photoelectron. Only the photoelectrons at the outermost surface can escape from the sample surface, making this a surface analysis technique.[115]

XPS analysis provides valuable information about surface layers or thin film structures within the range of many industrial applications including catalysis, corrosion, adhesion, polymer surface modification,[116] magnetic media, electronics, semiconductor, dielectric materials and thin film coatings packaging used in a number of industries.[117] The method is generally regarded as being the most quantitative, readily interpretable and most informative with regard to chemical information. Li and co-workers characterized the surface structure and composition of graft-modified and enzyme-functionalized polyaniline films using angle-resolved XPS. Subsequently, the binding energy and intensity of a photoelectron peak can be used to identify the elements contained in the sample surface.[118] The high-resolution spectra can establish the presence or absence of chemically distinct species (e.g. functional groups) [44] and quantify the chemical compositions (nitrogen, oxygen and carbon) on the surface of the sample.[119,120] The XPS curve fitting has been used to resolve components in the C 1s, N 1s and Br 3d regions while angle-dependent XPS provides vital information to assess the depth distributions and layer thicknesses of furanones.[121] Overall, a wealth of information furnished by XPS is useful to evaluate the efficiency of enzyme immobilization processes prepared using a multitude of immobilization techniques.[122]

### Surface plasmon resonance (SPR) by ultraviolet detection

Plasmon resonance is described as a collective oscillation of conduction band electrons that occurs with peak wavelengths that depend on the size, shape and material composition of the nanoparticle. Surface plasmon resonance (SPR) is a method used as a ruler of distance for dynamic biological processes whereby the biological plasmon coupling between noble metal nanoparticles can assess conformational changes up to approximately the diameter of the nanoparticle (>20 nm). The moving electrons generate an oscillating dipole that can couple with dipoles in other nearby nanoparticles.[123] Much larger shifts have been observed for Ag, but experimentally, Au is often used due to ease of functionalization and to work with in a solution.

SPR has been used directly to measure the maximal binding and equilibrium fractional surface coverage as the concentration of enzyme in a recirculating solution is stepped up and down in a manner similar to that of the stepwise surface titration method previously described for the study of protein–protein and protein–DNA interactions.[124] The surface coverage is plotted versus concentration as an adsorption isotherm to determine the equilibrium dissociation constant, *K*_d_, for the enzyme–vesicle interaction.[125] A label-free SPR can be employed to give a more accurate depiction of molecular interaction. For instance, when specific cytochrome P450 enzymes are attached to the alternate gold surface, SPR was used to distinguish between inhibitors and enzyme substrates based on the shift in absorbance wavelength. This is a feature particularly useful for any research studies that intend to ensure safety profile for potential drug candidates.[126,127]

### Circular dichroism (CD) spectroscopy

Circular dichroism (CD) spectroscopy is a powerful method in structural biology that has been used to examine proteins, polypeptides and peptide structures since the 1960s. It is a spectroscopic technique widely used for the assessment of the conformation and stability of proteins in several environmental conditions like temperature,[128,129] ionic strength/charge and presence of solutes or small molecules.[130] CD spectroscopy is non-destructive, relatively easy to operate, requires small amount of sample and small data collection. The technique uses a source of circularly polarized light, in which the vector oscillates rotationally to the right or to the left, forming a helix around the axis of propagation. To compare, when light is depolarized the electromagnetic vector oscillates in any direction perpendicular to the direction of propagation. When light is linearly polarized in a plane, the vector oscillates on a single plane in the direction of propagation.[131] CD spectroscopy is useful when probing the secondary, tertiary and the structural family of proteins as the peptide bond is asymmetric and shows the phenomenon of CD. The spectra of protein molecules in the far UV regions are dominated by the n π* and π π* transitions of amide, and are influenced by the geometries of the polypeptide backbones, their spectra are reflective of the different types of secondary structures present.[132] The amide chromophore of peptide bonds appears as CD spectra in regions below 250 nm, with two electronic transitions corresponding to n to π* and π to π* showing CD at 215–230 and 185–200 nm, respectively.[133,134] The tertiary structure of protein is characterized by π_o_ to π* transition observation of CD in the region between 250 and 300 nm. Although the aromatic residues largely contribute to the near-UV (>250 nm), they also contribute to the far-UV spectra of a protein. Overall, the contribution is very small but when the content of these residues is very high, the estimation of secondary structure becomes complicated. CD of disulphide bonds results from n to σ* transitions that occur at approximately 260 nm, characterized by a wider peak than that of an aromatic residue.[131] CD spectroscopy is particularly helpful in enzyme immobilization studies by providing structural information with reference to protein–ligand interactions and protein–protein interactions,[132–134] structural compositions of proteins, kinetic [135] and thermodynamic information about macromolecules can also be derived from CD spectroscopy.

### Atomic force microscopy (AFM)

Atomic force microscopy (AFM) is the only microscopic technique able to visualize biomolecules at the single-molecule level with sub-nanometre accuracy in liquid.[136,137] Hence, the technique is widely used in many surface-based protein–protein interactions, biosensors, single-molecule analysis, bioelectronics or drug screening.[96,137] AFM basically allows visualization of the surface topography of enzyme molecules on the surface of the carrier,[16,96] adhesion, elasticity, association processes, dynamics and other properties of biological samples.[138] AFM can also provide information on protein–protein and protein–surface interactions, through force spectroscopy measurements, and on surface-induced conformational changes of enzymes.[137,139]

The AFM probe consists of a microfabricated cantilever which tapers into a sharp nanotip that can be moved in three dimensions with sub-nanometre accuracy by means of several piezoelectric scanners. The tip is brought near the sample surface so that forces acting on the tip cause the cantilever to bend. A laser beam is aimed at the top of the cantilever and reflected onto a photodiode. By attaching one of the interacting molecules to the AFM tip and the other molecule to the sample surface, the molecular binding forces can be quantified from the positive binding/rupture events.[138]

The AFM system has two modes of operation which includes single-molecule force spectroscopy (SMFS) and jumping mode (JM). In the SMFS, the cantilever deflection is recorded as a function of the vertical displacement of the piezo scanner to quantitatively analyse ligand–receptor interactions to reveal the nature and magnitude of forces, and the related binding energy landscape. The force-scan-based JM is able to obtain a simultaneous topography assessment and also quantify the unbinding [140] between receptor molecules on a sample and a ligand using tip–sample adhesion maps obtained through the attached AFM tips.[141] AFM was used to assess the enzymatic activity of the immobilized glutamate dehydrogenase molecules on the biosensor surface. In another study, fractal dimension of the immobilization sensor surface was used as a parameter to evaluate the quality of the immobilized biosensors [142] as well as to assess the controlled and oriented immobilization of ordered monolayers of enzymes prepared using a novel method.[137]

### Microcalorimetry

The term calorimetry is defined as a measurement of the relationship of the change of temperature according to time during the process of program-controlled temperature. Microcalorimetry is a versatile technique for studying thermal activities in terms of heat, heat flow and heat capacity. An expected thermal power can be calculated from analytical data describing net changes of the reaction system together with their molar or specific enthalpy values which normally are known (‘indirect calorimetry’) and be compared with results of a direct calorimetric determination.[143] The technique can precisely address two primary questions about immobilized proteins: (1) how does the stability of the protein change after immobilization; (2) how was the biological activity affected?[144] The technique can be completely non-destructive and non-invasive to the sample as it seldom requires any prior sample treatment nor does it limit analysis to a physical state of the sample. Samples in the form of solids, liquids and gases can all be investigated.[145] Calorimetric analysis can provide direct information on the stability and biological activity of immobilized proteins. It works on the principle that all physical and chemical processes are accompanied by a heat exchange with their surroundings. When a reaction occurs a temperature gradient is formed between the sample and its surroundings to result in heat flow between the sample and the surrounding that is measured as a function of time.[146]

Different calorimetric techniques can be adapted to investigate the variable aspects of the protein chemistry, depending on the physical environment and the type of confinement. In differential scanning calorimetry experiments, the thermodynamic parameters such as the middle point temperature and enthalpy change of the unfolding transition of either the immobilized or free protein can be obtained. The technique is fairly flexible to provide insight on the thermodynamic effects of the immobilization, as such from multipoint covalent attachment to simple absorption [144] testing of protein stability, DNA–drug binding studies, protein formulation stability, stability of sutant and wild-type proteins, membrane and lipid stability and polynucleotide stability.[147,148] Isothermal batch and flow calorimetry can assess the effects of the immobilization, support environment and the type of entrapment on the active site by measuring the differences in the binding capacity of specific ligands. Both techniques are suitable to establish reaction enthalpy changes and equilibrium constants from full protein–ligand titration curves.[144]

### Forster resonance energy transfer (FRET)

Forster resonance energy transfer (FRET) is a quantum mechanical phenomenon that occurs between two fluorescent molecules. It is a distance-dependent physical process, by which non-radiative energy is transferred from an excited molecular fluorophore (the donor) to another fluorophore (the acceptor) through intermolecular long-range dipole–dipole coupling.[149–151] Accurate measurement of molecular proximity at angstrom distances (10–100 Å) is possible and the technique is highly efficient if the donor and acceptor are positioned within the Forster radius (the distance at which half the excitation energy of the donor is transferred to the acceptor, typically 3–6 nm). FRET only needs one of the two molecules to be fluorescent and the distance between the donor and acceptor is kept to minimum to ensure high probability of energy transfer. The donor and acceptor fluorophores must be adequately aligned for proper induction of the acceptor dipole by the donor.[152,153] The precise way in which these molecules interact allows the emission spectrum of a donor fluorophore to significantly overlap (>30%) the absorption spectrum of an acceptor [149,152] that made molecular scale measurements feasible.[153] Hence, the technique has been referred to as ‘the spectroscopic ruler.’[154]

The use of FRET between fluorophores has been enhanced [151,155] by the introduction of new generation fluorophores which include small and photostable organic fluorophores as well as activated nanoparticles that were brought in as a substitute to the native fluorophores, such as tryptophan and green fluorescent protein. The method requires covalent attachment of a FRET pair, the donor fluorophore and acceptor dye at specific sites of the biomolecules [152,156] to produce the overlapping emission peak of the donor and the excitation peak of the acceptor. The sample is irradiated at the absorption wavelength of the donor which is temporarily excited into a higher energetic electronic state. The donor fluorophore then gives up its energy non-radiatively to the acceptor fluorophore by dipole-induced dipole interaction and decay at its characteristic fluorescence emission wavelength. The emission wavelengths of both donor and acceptor are monitored to quantify the efficiency of energy transfer between the donor and acceptor.

Measurements of FRET efficiency have long been used at the ensemble level to monitor conformational changes of molecules in solution [154] such as examining structural and dynamic properties of individual molecules on an atomic level, analysis of molecular interactions at the level of single cells, cell organelles[157,158] and single molecules including time trajectories of folding pathways and transient intermediates of enzymes when immobilized.[159] Also, analysis of biological reactions and the characteristics of freely diffusing or immobilized biomolecules *in vitro* and living cells [160,161] is made possible with FRET and is now a standard tool for nanometre scale investigation of inter- and intramolecular distances.

FRET offers the benefit of a practical and simple measurement for cases in which the main objective is to distinguish between two molecular states with different donor–acceptor distances.[127] However, FRET-based quantitative measurements of distance demand a variety of experimental factors, including different excitation and detection efficiencies for the donor and acceptor dyes and differences in transfer efficiency based on fluorophore orientation and chemical environment. Hence, these factors account for biases due to (1) bleed-through in excitation, such as when a donor is excited by the acceptor's excitation wavelength and vice versa; and (2) crosstalk in emission detection, such as when the emission of a donor also contributes to the signal measured in a set-up for acceptor detection, and vice versa. It is often difficult to separate the contribution of direct crosstalk from the contribution of bleed-through signals.[151]

### Scanning electrochemical microscopy (SECM)

Scanning electrochemical microscopy (SECM) was invented by Bard and co-workers in 1989 and it is an instrument which basically consists of a combination of electrochemical components, positioners and computer control. Current is allowed to flow through a microelectrode immersed in an electrolytic solution and situated close to a substrate. The microelectrode and the substrate form part of an electrochemical cell which is also constituted by reference and auxiliary electrodes, and sometimes by a second working electrode.[162] The substrate can either be a conductive, semiconductive or insulating material. An ultramicroelectrode called a tip is used to scan a surface of interest in close proximity. The electrochemical response of the tip or of the substrate in response to the tip provides quantitative information about the interfacial region.[163]

SECM has been used for the quantitative investigation and surface analysis of a wide range of processes that occur at interfaces [164] and for probing a great variety of electrochemical processes in fundamental and applied electrochemistry,[165] energy storage,[166] materials science,[167] corrosion science, biosensors research [168] and biophysics.[169] The technology was used to investigate transport properties inside modified layers in feedback mode [170]; this analysis offers a characterization of the modified surface and the measurement of the enzymatic activity depending on the concentrations of glucose and mediator.[171] SECM is a virtually new electrochemical technique [165,172–174] that has shown great promise for the studies of immobilized biomolecules and, chemical and biological reactions at the electrode–solution interface.[174–179] SECM can also be used to detect redox catalysis displayed by an immobilized enzyme and measure the apparent kinetics of the surface-catalysed electron-transfer reaction.[175] Takenaka and co-workers reported on SECM imaging of surface-confined DNA molecules and DNA hybridization in a highly localized surface area [180–182] which allows direct visualization of DNA microarrays with the aid of an electroactive hybridization indicator.[182]

### Time-of-flight secondary ion mass spectroscopy (TOF-SIMS)

The time-of-flight secondary ion mass spectroscopy (TOF-SIMS) is a surface-sensitive analytical tool that permits the submicron-scale mapping of complex sample surfaces such as protein-adsorbed materials, and chemical mapping information, such as the imaging of the distribution pattern of a particular protein.[183] It is a powerful surface characterization technique that is able to provide detail surface characterization including the composition, structure, orientation and spatial distribution of the molecules and chemical structures on the surface.[184]

The components of TOF-SIMS consist of an ultrahigh vacuum system, a particle gun that uses Ga or Cs source, a circular designed flight path equipped with electrostatic analysers and a mass spectrometer that utilizes TOF analyser to enhance its sensitivity and increase its range of application.[185] The method uses a pulsed beam of primary ions which bombards the surface and removes molecules from its very outermost of a sample, which are emitted in a variety of types of secondary particles, such as photons, neutrons, secondary electrons as well as positive and negative secondary ions.[183] The secondary ion particles removed from atomic monolayers on the surface are then accelerated to a TOF mass analyser at a given potential, *V* (2–22 keV) and all ions with the same electric charge have the same kinetic energy in the TOF analyser. The ions are allowed to travel through a path of a given length for a certain time span before reaching the detector. Since the velocity of each secondary ion is dependent on its weight, the mass of the ions is determined by measuring the exact time at which the ions reach the detector. Depending on the mass to charge ratio, generally ions that are lighter will reach the detector quicker.[183]

The TOF-SIMS is available in three modes operating, namely, surface imaging and surface spectroscopy, for which both are used for visualization of distribution of individual species on a surface and analysis of elemental and molecular species on a surface, and depth profiling for determination of different chemical species as a function of depth from the surface. TOF-SIMS has been already used in the steric analysis of proteins,[16] lipid–lipid and lipid–protein interactions,[186,187] biomarkers [188] as well as enzyme immobilization.[189] For the purpose of characterization of organic samples, the less damaging TOF-SIMS static measurement mode is used as the samples are sputtered softly with ions with low current densities that leave the sample structure intact. The dynamic mode is preferred when extreme high sensitivity analysis is required in which ions of high current densities are used to bombard the sample surface, which in tern leads to damaging the surface. This measurement method is more appropriate for analysis of inorganic materials such as trace elements.[183,185,190]

The sampling depth of TOF-SIMS is less than 2 nm, whereas the size of most proteins is larger. Hence, this method provides the chemical structure of the surface side of an immobilized enzyme representing the enzyme orientation. The method is appropriate for highly sensitive detection as it provides extremely high transmission in combination with parallel detection of all masses.[191] Biomaterials including protein can be quantitatively measured and the distribution of the molecules of interest can also be established. However, TOF-SIMS has till now not come into widespread use in the field of enzyme immobilization because of its complicated spectrum interpretation.[16,192]

## Conclusion

Enzyme-based strategies are increasingly favoured over the conventional chemical methods in industrial processes. Utilization of immobilized enzymes as biocatalysts will continue to attract significant attention from industries as the technique is highly efficient, environmentally friendly and can potentially be cost saving when further research is done to seek out or manufacture new matrices that are cheaper and more robust, which can be used as supports for enzyme immobilization. Also, more studies should be centred to overcome the current drawbacks in immobilization techniques, as well as development of simple and stable enzyme immobilization methods that could bring down the cost of immobilized enzymes.

The present surface analytical technologies are useful for monitoring efficacy of an immobilization process as well as to keep track of post-immobilization changes in the enzyme. These techniques provide vital insights into the effects of immobilization of enzyme on the stability and activity following treatment with different immobilization methods. In time, these modern techniques which combine tools from chemistry and molecular biology can be further developed to help improve enzyme immobilization strategies and expand the catalytic repertoire of immobilized enzymes for diverse application in various fields.
